# Modulation of early gene expression responses to water deprivation stress by the E3 ubiquitin ligase ATL80: implications for retrograde signaling interplay

**DOI:** 10.1186/s12870-024-04872-5

**Published:** 2024-03-08

**Authors:** Manuel Méndez-Gómez, Daniel Sierra-Cacho, Estela Jiménez-Morales, Plinio Guzmán

**Affiliations:** Departamento de Ingeniería Genética, Centro de Investigación y de Estudios Avanzados del IPN, Unidad Irapuato, Irapuato, 36824 Gto México

**Keywords:** Water deficient stress, Primary response genes, E3 ubiquitin ligase, Cistrome, Retrograde signaling

## Abstract

**Background:**

Primary response genes play a pivotal role in translating short-lived stress signals into sustained adaptive responses. In this study, we investigated the involvement of ATL80, an E3 ubiquitin ligase, in the dynamics of gene expression following water deprivation stress. We observed that *ATL80* is rapidly activated within minutes of water deprivation stress perception, reaching peak expression around 60 min before gradually declining. *ATL80*, despite its post-translational regulation role, emerged as a key player in modulating early gene expression responses to water deprivation stress.

**Results:**

The impact of ATL80 on gene expression was assessed using a time-course microarray analysis (0, 15, 30, 60, and 120 min), revealing a burst of differentially expressed genes, many of which were associated with various stress responses. In addition, the diversity of early modulation of gene expression in response to water deprivation stress was significantly abolished in the *atl80* mutant compared to wild-type plants. A subset of 73 genes that exhibited a similar expression pattern to *ATL80* was identified. Among them, several are linked to stress responses, including ERF/AP2 and WRKY transcription factors, calcium signaling genes, MAP kinases, and signaling peptides. Promoter analysis predicts enrichment of binding sites for CAMTA1 and CAMTA5, which are known regulators of rapid stress responses. Furthermore, we have identified a group of differentially expressed ERF/AP2 transcription factors, proteins associated with folding and refolding, as well as pinpointed core module genes which are known to play roles in retrograde signaling pathways that cross-referenced with the early ATL80 transcriptome.

**Conclusions:**

Based on these findings, we propose that ATL80 may target one or more components within the retrograde signaling pathways for degradation. In essence, ATL80 serves as a bridge connecting these signaling pathways and effectively functions as an alarm signal.

**Supplementary Information:**

The online version contains supplementary material available at 10.1186/s12870-024-04872-5.

## Background

Cells respond to signals activating primary and secondary transcriptional programs. During primary responses, expression of an array of genes is induced within minutes, independently of de novo protein production; these genes are known as primary response genes (PRG)s. Secondary response genes are subsequently expressed that require de novo protein synthesis, and these genes are more numerous than primary response genes, which serve as master switches from brief and transient signals to extended responses [1]. Mammalian proto-oncogenes c-fos and c-jun are prototypes of primary response genes which are implicated in cell proliferation and differentiation in response to diverse stimuli (i.e., developmental and stress signals, growth factors, cytokines, xenobiotics) [2]. c-fos is among various primary response genes that are transiently expressed in neurons within minutes after synaptic activation [3]. Conversely, c-fos expression is constitutive and not regulated in many cancer cells [4].

In plants, many developmental and cellular processes and responses to physical injury and abiotic and biotic stresses are triggered by primary response genes. Auxin action guides many growth and development processes through the transcriptional regulation of a short signaling pathway. Auxin induces the expression of primary response genes within minutes, including members of the Auxin/Indole-3-Acetic Acid (Aux/IAA) and the auxin response factor (ARF) families. Members of the Aux/IAA and ARF families interact between them, generating a group of diverse dimers that function as transcriptional repressors routing many auxin responses [5]. The primary response cytokinin response genes comprise the type-B Response Regulator (RRBs) transcriptional factors. Transcription factor (TF) complexes originated by homo- and heterodimerization of RRBs and by interaction with other TF (i.e., DELLA proteins, EIN3, HD-ZIII, BZIP63, SPL9) mediate the response to this phytohormone. Both plants and animals have preserved an innate immune system during evolution to respond to tissue injury and pathogen attack. Cell surface receptors interact with foreign molecules, including endogenous damage-associated molecular patterns (DAMPs) and microbe- or pathogen-associated molecular patterns induce (MAMPs/PAMPs) to trigger strong defense responses. Primary response genes are essential components of these responses, with the perception of DAMPs and MAMPs/PAMPs leading to the activation of many primary immune response TFs and signaling components. These pathways of ten converge on regulatory mitogen-activated protein kinase (MAPK) cascades [6].

Plant-specific A-Type RING Ligases (ATLs), formerly known as Arabidopsis Tóxicos en Levadura, make up a large family of RING-type ubiquitin-ligases. They emerged around 470 million years ago when plants colonized land. *Arabidopsis thaliana*, for example, has 100 ATLs [7]. These ATLs are key players in the ubiquitination pathway, interacting with ubiquitin and ubiquitin-conjugating enzymes (E2s) to mark target proteins for degradation. A typical ATL comprises a RING-H2 finger for interaction with E2 conjugases, transmembrane helices at the N-terminus for directing ATLs to specific cellular compartments, and a conserved 12–16 amino acid motif known as GLD, located between the hydrophobic domain and the RING-H2 [7].

*ATLs* are frequently involved in plant immunity and responses to environmental stresses. For instance, *ATL2* is rapidly and transiently activated in response to pathogen-associated molecular patterns (PAMPs), ATL9 is involved in the expression of defense-associated genes, cell death, and callose deposition, and ATL12 contributes to resistance against fungal pathogens [8, 9]. A pair of closely related *ATL* paralogs, *ATL6* and *ATL31*, and *ATL44*/*RHA3A* and *ATL45*/*RHA3B* mediate the homeostasis of BOTRYTIS-INDUCED KINASE 1 (BIK1), a central component regulating immune responses. ATL6 and ATL31 facilitate BIK1 stability by targeting CALCIUM-DEPENDENT PROTEIN KINASE28 (CPK28), a regulator of BIK1 turnover, and ATL44/RHA3A and ATL45/RHA3B mediate the monoubiquitination of BIK1 which is essential to boost immune responses [10, 11]. ATLs have been shown to grant tolerance to environmental stresses. For instance, ectopic expression of ATL61 enhances drought tolerance in Arabidopsis, and the sweet potato IbATL38 improves salt stress tolerance [12, 13]. In rice, decreased expression of OsATL38 enhances tolerance to cold stress through mono-ubiquitination of the 14-3-3 protein OsGF14d [14].

*ATL78* orthologs are implicated in tolerance to drought stress in *A. thaliana* and tomato. In *A. thaliana*, an *atl78* mutant shows reduced tolerance to drought stress; consistently, in tomato, the ectopic expression of *ShATL78* enhances tolerance to drought [15, 16]. Interestingly, *ATL78* was linked to an adaptation to drought tolerance during the evolution of Brassicaceae. As a result of a segmental duplication event, *ATL81* with diminished expression generated *ATL78*, a paralog with an enhanced expression that conferred a tolerance phenotype [17]. Among *ATLs* whose expression is induced in drought stress, we identified *ATL80*, which shows features of a primary response gene that its expression following water deprivation stress (WDS) [18]. To understand the impact of ATL80 on differential gene expression, a time-course microarray experiment was conducted to compare responses to WDS between wild-type and *atl80* mutant plants. The results revealed that ATL80 significantly influenced gene expression, leading to a severe decrease in differentially expressed genes (DEGs) in the *atl80* mutant compared to the wild type. Additionally, among the DEGs, components common to the retrograde signaling pathways were identified, suggesting the existence of converging signaling pathways.

## Methods

### *Arabidopsis thaliana* growth conditions and water deprivation treatments

We utilized WT Arabidopsis thaliana Col-0 and the mutant *atl80* (bearing a T-DNA insertion SALK_046204) for our research. The SALK_046204 mutant line was acquired from ABRC, genotyped using the SIGnAL iSect Toolbox (http://signal.salk.edu/isects.html), and underwent backcrossing prior to analysis. The T-DNA insertion in SALK_046204 disrupts the coding sequence of the intronless *ATL80* gene, and this insertion is expected to result in a severe loss-of-function phenotype. The plants were cultivated under controlled environmental conditions with a 16-hour light/8-hour dark cycle at 23 °C. In vitro growth conditions for both WT Col-0 and the *atl80* mutant were conducted following previous protocols. Approximately 25 seeds from each Arabidopsis WT Col-0 and *atl80* were surface-sterilized and placed on homemade rafts made of fabric (polyester and rayon) positioned on a polypropylene mesh supported by a sponge. These rafts were then positioned inside Magenta^™^ boxes containing 30 ml of liquid MS medium and 0.5% sucrose. The seeds were subjected to stratification for four days in darkness within a cold room and were subsequently incubated for 13 days in a growth chamber with a 16-hour light/8-hour dark cycle at 23 °C. At the conclusion of the incubation period, the seeds were transferred to new Magenta boxes containing MS medium without sucrose and incubated for an additional five days. On Day 5, the rafts were removed, placed on filter paper, and exposed to continuous air dehydration for various time intervals in a laminar flow hood, including 0, 15, 30, 60, and 120 min. For experiments involving mature plants, the plants were watered every 4 days for 20 days following germination (hydrated condition). This was followed by a suspension of irrigation for 15 days (dehydration condition), and then rewatering for 1 day (rehydrated condition). Cycloheximide (SIGMA) treatment was administered to eighteen-day-old WT seedlings submerged in MS medium supplemented with 70 μm cycloheximide for 0, 15, 30, 60, and 120 min.

### Determination of relative fresh weight, chlorophyll content, and reactive oxygen species following water deprivation

Physiological experiments were performed in WT and *atl80* mutant plants grown in vitro in a growth chamber for 13 days and then for five more days in media without sucrose, as described above. For each dehydration time point (0, 15, 30, 60, and 120 min), 100 mg of tissue from the seedlings was collected and weighed. Excess liquid media was briefly blotted on filter paper, and samples were weighed immediately. Two replicas were collected for each time point. Fresh weight (FW) was obtained by collecting and immediately weighing the samples after removing excess water.

To quantify total chlorophyll (TCh) content, WT and *atl80* seedlings for each time point were suspended in 96% ethanol and kept in the dark for 1 h before they were ground and homogenized with 2.5 ml 80% acetone in 5 mM sodium phosphate buffer (pH 7.5). The supernatant was collected and placed in a glass cuvette that was placed in a spectrophotometer to measure the absorbance at 663 and 645 nm (A645 and A663, respectively). The readings were used to determine the TCh concentration with the following equation: TCh (µg ml^− 1^) = 5.24A_664.2_ + 22.24A_648.6_; as previously described. To analyze reactive oxygen species, a hydrogen peroxide kit (National Diagnostics, Atlanta, GA, USA) was used. WT and *atl80* mutant seedlings were ground into a powder that was dissolved in 500 µl Milli-Q water. The mixture was stirred for 20 min and then centrifuged for 15 min at 12,000 g. Aliquots (10 µl) of each sample were mixed with 90 µl of the kit solution (ferrosol iron + xylenol orange) and incubated at room temperature for 30 min. Then, the absorbance was read at 560 nm and the H_2_O_2_ concentration was calculated from a standard curve.

The data were analyzed using the STATISTICA program version 12.5. One-way and two-way ANOVA analyses were conducted, followed by a post hoc test (Tukey’s HSD) to examine differences between groups. Significance levels were determined at *P* < 0.05, and distinct letters were employed to indicate statistically significant differences (Tukey’s HSD, *P* < 0.05).

### Drought transcriptome profiling

WT and *atl80* seedlings from each time point were collected for control and dehydrated conditions. Two biological replicas were obtained from pools of seedlings grown under the same conditions. RNA was isolated from frozen seedlings using a RNeasy plant mini kit (Qiagen; Hilden, Germany). Labeling of total RNA, microarray processing data analysis, and normalization was performed by Oaklabs GmbH (Hennigsdorf, Germany). An ArrayXS Arabidopsis v2 (XS-5010) microarray in the Agilent 8 × 60 K format representing 30,541 *Arabidopsis* genes was used. TAIR9 release was used to annotate the genes, as provided by Oaklabs. Subsequently, TAIR10 and/or Araport111 were used to annotate all differentially expressed genes as defined by comparison of the *atl80* mutant transcriptome with that of WT under each of the three conditions. P-values and log2 fold-changes were calculated among the groups of replicate samples. To calculate P-values, a two-tailed Student’s t-test was performed. The P-value cutoff was generally at 5% (calculated by two-tailed Student’s t-test) with an absolute log2 fold-change ≥ 1.7.

### qRT‑PCR analysis

RNA was extracted from frozen seedlings using the RNeasy Plant Mini Kit (Qiagen, Hilden, Germany). cDNA synthesis was carried out with 3 µg of total RNA using the SuperScript III First-Strand Synthesis SuperMix for qRT-PCR (Invitrogen). Quantitative RT-PCR was conducted on a CFX96 Real-time System (Bio-Rad, Hercules, CA, USA) with Maxima SYBR Green qPCR Master Mix (2X) and analyzed using CFX Manager software version 3.1 (Bio-Rad, Hercules, CA, USA). The PCR conditions consisted of an initial denaturation at 95 °C for 3 min, followed by 40 cycles of 95 °C for 15 s and 63 °C for 30 s. All qRT-PCR experiments were performed with two biological replicates and three technical replicates. To normalize the data, the expression of the reference genes AT3G18780 (ACTIN2) and AT1G13440 (GAPC2) was used.

### Gene Ontology (GO) term enrichment

For a comprehensive appraisal of GO term enrichment, we introduced more suitable GO classes. We categorized differentially expressed into five classes and into a class termed “other components” (a class that included non-categorized genes). The five classes were the 43 categories to assist the identification of archetypal genes from each time point are as follow: The six classes and 43 categories (in parentheses) are: gene expression (basic transcriptional machinery, maintenance of the genome/chromatin organization and remodeling, non-coding RNA, posttranscriptional regulation, TFs, tRNA and translational machinery), regulation of signaling (calcium signaling and binding, dephosphorylation, ion/biomolecule binding, lectin gene family, leucine-rich repeat, peptides, phosphorylation, protein-protein interaction, receptor-like protein kinase, ubiquitination), growth and development (senescence, cell cycle and cytoskeleton, circadian clock, hormones, modulator of growth, reproductive development, senescence, tropism/hydrotropism), metabolism (glucosinolate metabolism, lipid metabolism, nutrition, oxidation-reduction process, photosynthesis/light response/chloroplast function, primary metabolic process, secondary metabolism, endocytic pathway, transport), protection (cell wall and membrane dynamics, dehydration/hypoxia/freezing tolerance, detoxification, lipid transfer, proteases, protein folding/refolding, response to stress factors, response to stress factors-abiotic, response to stress factors-biotic, sugar and osmolytes, water transport).

### *cis*-regulatory element enrichment on promoters of PRGs

The cis-element enrichment analysis was performed using the CisCross web service (https://plamorph.sysbio.ru/ciscross/). The analysis aimed to predict upstream regulators, and the following parameters were employed: promoter length, 1500 bp; background, ARAPORT11; DAP-seq collection, Plant Cistrome, CisCross MACS2; multiple comparisons, Benjamini-Hochberg; adjusted p-value threshold, 0.001 [19, 20].

## Results

### Influence of *ATL80* on the early response to water deprivation stress

**Fig. 1:**
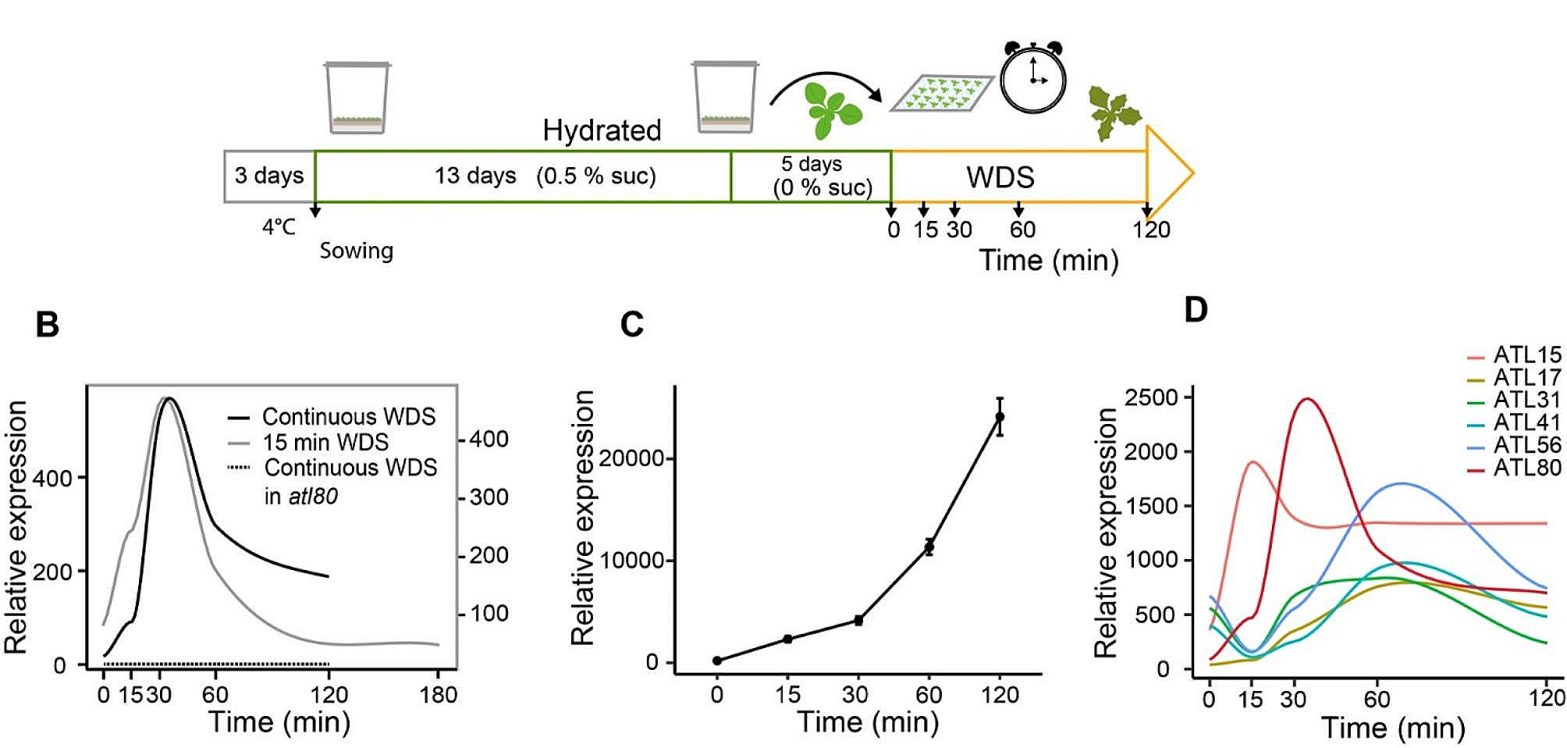
Influence of ATL80 on the early response to WDS. **(A)** Experimental design to inspect the impact of ATL80 on the initial response to continuous WDS. Eighteen-day-old WT and *atl80* seedlings, cultivated in vitro, were exposed to continuous air dehydration in a laminar flow hood. Explants were collected at 0, 15, 30, 60, and 120 min. The expression of *ATL80* was also evaluated in the *atl80* mutant insertional line (dashed line). The details of the experimental setup can be found in the Methods section. **(B)** Transcriptional response of *ATL80* to WDS. For the 15 min response to WDS, data was obtained from the AtGenExpress project (dark line) [18]. The continuous WDS response was conducted as described in (A) and quantified using real-time quantitative PCR (pPCR) (gray line). **(C)** Effect of the translational inhibitor cycloheximide on *ATL80* expression. Eighteen-day-old seedlings WT were incubated with 70 μm of cycloheximide for 0, 15, 30, 60, and 120 min and relative expression was evaluated by qPCR. **(D)** Evaluation of the expression levels of early responsive ATLs to WDS among the 100 *A*. *thaliana* ATLs. Genes displaying enhanced early expression in response to WDS are shown

Stress induced by water deficit significantly hampers plant growth and development. In response to this stress, a multitude of changes in gene expression takes place. Most investigations focusing on alterations in gene expression have historically involved prolonged exposure to drought stress [21]. In our examination of ATL-type E3 ligases involved in drought stress within community databases for Arabidopsis, we identified an interesting observation. In the AtGenExpress abiotic stress microarray analysis, *ATL80* exhibited a rapid and transient response to water loss, suggesting that *ATL80* acts as a primary response gene [18]. The drought experiments conducted as part of the AtGenExpress project aimed to assess the impact of a 15-minute water deficit stress (WDS) exposure on gene expression. Seedlings grown in liquid media within Magenta boxes were removed and exposed to an air stream for 15 min. Subsequently, they were returned to the Magenta boxes, incubated in the growth chamber, and explants were collected after 15, 30, 60, 180, and 360 min [18]. In a bid to investigate the effect of continuous WDS on early gene expression under similar conditions, we designed an experiment (Fig. 1A). Our findings revealed a swift and transient transcriptional response of *ATL80* when seedlings were subjected either to WDS for 15 min or continuous WDS. *ATL80* expression peaked after 30 min of WDS and subsequently decreased after 90 min (Fig. 1B). Remarkably, no other biotic or abiotic stimuli tested elicited such a transient response (Kilian et al., 2007). Notably, *ATL80* expression exhibited a substantial increase even in the absence of new protein synthesis, which aligns with the characteristics of primary response genes. This induction of *ATL80* expression was notably pronounced in the presence of the translational inhibitor cycloheximide (Fig. 1C).

To contextualize the expression of *ATL80*, we compared it to other *ATLs* that exhibited early expression in response to WDS. *ATL80* displayed an expression pattern reminiscent of a primary response gene. *ATL15* exhibited an initial increase at 15 min but showed a minor decline thereafter. In contrast, *ATL17*, *ATL31*, *ATL41*, and *ATL56* showed a peak of expression after 60 min, and, with the exception of *ATL17*, their expression initially decreased in the first 15 min after WDS before peaking (Fig. 1D). Based on these observations, we postulated that ATL80 might play a role in modulating early transcriptional responses of plants to dehydration.

**Fig. 2:**
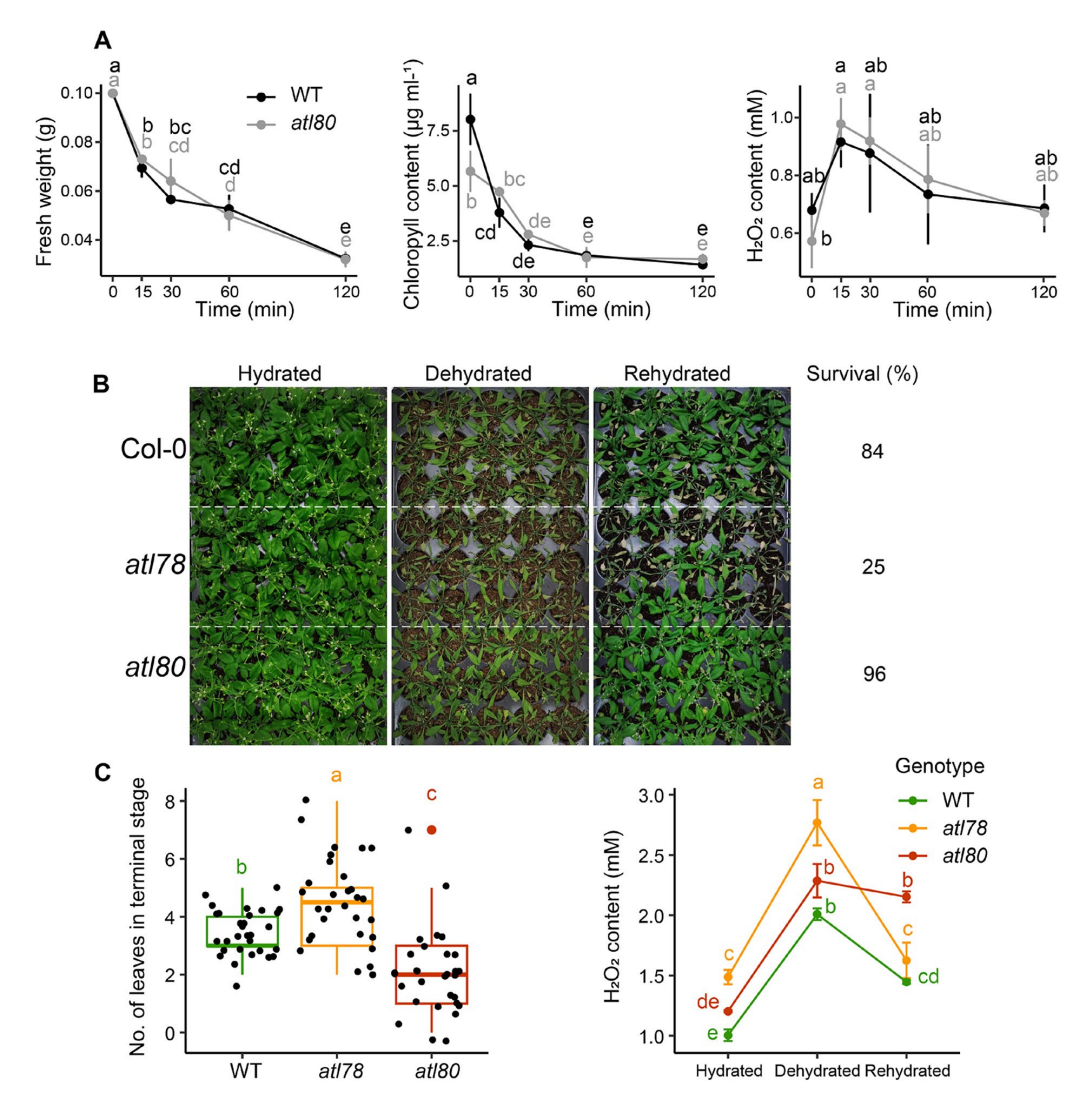
Impact of ATL80 on standard responses to WDS. **(A)** Comparison of WT (dark line) and the *atl80* (gray line) mutant on relative water content, total chlorophyll content, and H_2_O_2_ content during the time course experiment in seedlings. **(B)** Survival rates of WT, *atl78*, *atl80* lines after WDS. Plants were watered every 5 days for 20 days after germination, followed by suspension of irrigation for 11 days, and then rehydrated for one day. Survival percentages were recorded one day after rehydration. **(C)** Number of leaves in the terminal stage of senescence (chlorotic leaves) recorded after rehydration (left panel), and H_2_O_2_ content (right panel) obtained from the experiment in **(B)**

The *atl80* mutant did not exhibit noticeable disruptions in basic physiological processes associated with WDS. This was evident from the similar response observed in both wild-type (WT) and mutant lines, which manifested as a progressive decrease in fresh weight, chlorophyll content, and H_2_O_2_ production (Fig. 2A). In an attempt to identify a water stress-related phenotype in *atl80*, adult plants were subjected to a dehydration/rehydration experiment. Seedlings were watered every 4 days for 20 days after germination, followed by a suspension of irrigation for 15 days, and then rewatered for one day. After rehydration, it was observed that 84% of WT plants and 23% of the control *atl78* survived, whereas 96% of the *atl80* mutant did, suggesting a moderate effect of ATL80 on the survival of adult plants after rehydration (Fig. 2B). We also determined the number leaves in the terminal stage of senescence and H_2_O_2_ production. The number of chlorotic leaves is lower in *atl80* compared to WT and *atl78* plants (Fig. 2C, left panel). During dehydration, there was a progressive increase in H_2_O_2_ production observed in all three lines. However, upon rehydration, H_2_O_2_ production significantly decreased in the WT and in the *atl78* mutant, and to a somewhat lesser extent in the *atl80* mutant. Additionally, the *atl80* mutant displayed an accelerated flowering time, indicative of a higher growth rate [22].

### Time course microarray analysis of primary response genes in wild-type and the *atl80* mutant after WDS

**Fig. 3:**
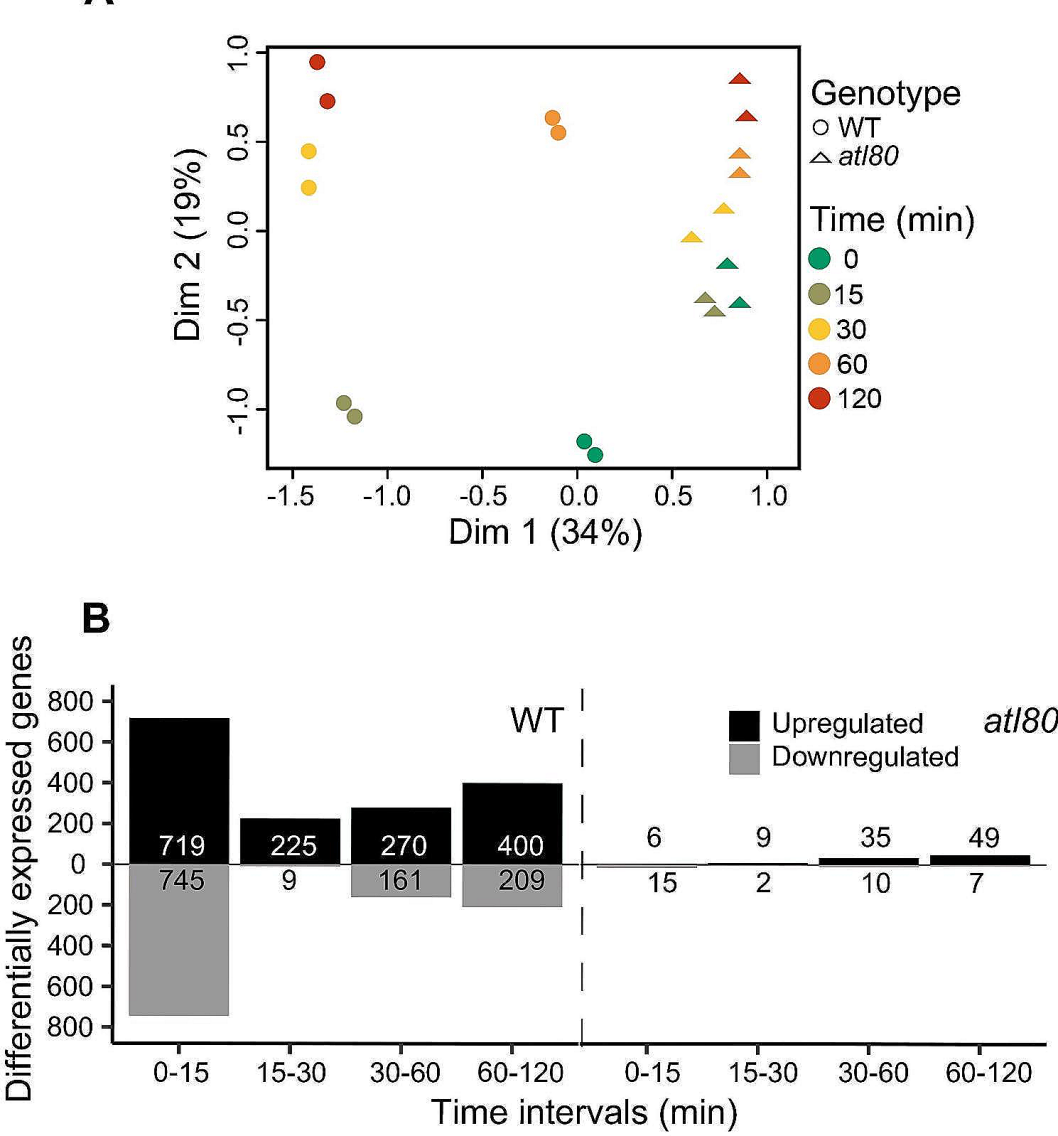
Influence of *ATL80* on the transcriptome in response to WDS. **(A)** Principal Component Analysis (PCA) of Raw Expression Data. PCA was performed on the raw expression data of all samples. Dots represent WT samples, color-coded for easy distinction, while triangles represent atl80 mutant samples. The axes represent the principal components. **(B)** Comparison of WT and *atl80* transcriptomes between time points. The number of DEGs in WT and *atl80* are presented in the left and right panels, respectively

The expression of *ATL80* exhibits remarkable dynamics in response to WDS, undergoing rapid changes within minutes. We reasoned that gene expression changes dependent on ATL80 might occur during these brief time intervals, and that these changes could be readily detected than alterations in protein stability regulated by the E3 ubiquitin ligase activity of ATL80. Accordingly, as an initial step in analyzing ATL80 function we conducted a time course microarray analysis experiment spanning 0, 15, 30, 60, and 120 min to assess the influence of ATL80 on the early transcriptome in response to WDS. Its impact on early gene expression in response to WDS. We employed an ArrayXS Arabidopsis chip containing 32,073 genes for this purpose. The transcriptome time-course after exposure to WDS was profiled using “in vitro” grown seedlings, and two biological replicates were included in the analysis (Fig. 1A and Methods). Principal component analysis (PCA) conducted on the raw data from all samples shows the biological variation among sample replicates and distinguishes differences between treatments and genotypes. The PCA analysis revealed that the transcriptional profiles of the replicates were highly similar, whereas significant differences emerged at various time points, primarily at 15 and 30 min after dehydration in the WT. Interestingly, it becomes evident that the *atl80* mutant fails to exhibit a response to dehydration, given the similarity observed across different time points. This dissimilarity between the WT and the mutant plant underscores the important role of ATL80 in the initial response to dehydration (Fig. 3A). Differentially expressed genes (DEGs) were identified for both WT and the *atl80* mutant at various time points. The results indicated that ATL80 had a significant impact on gene expression, with a striking 95% reduction in DEGs observed in the *atl80* mutant compared to the WT (Fig. 3B). Notably, nearly 50% of these DEGs were detected in WT plants within the first 15 min of exposure to WDS. During this initial 15-30-minute interval, over 95% of DEGs exhibited upregulation.

### Dynamic bursts of differentially expressed genes between WT and *atl80* transcriptomes

**Fig. 4:**
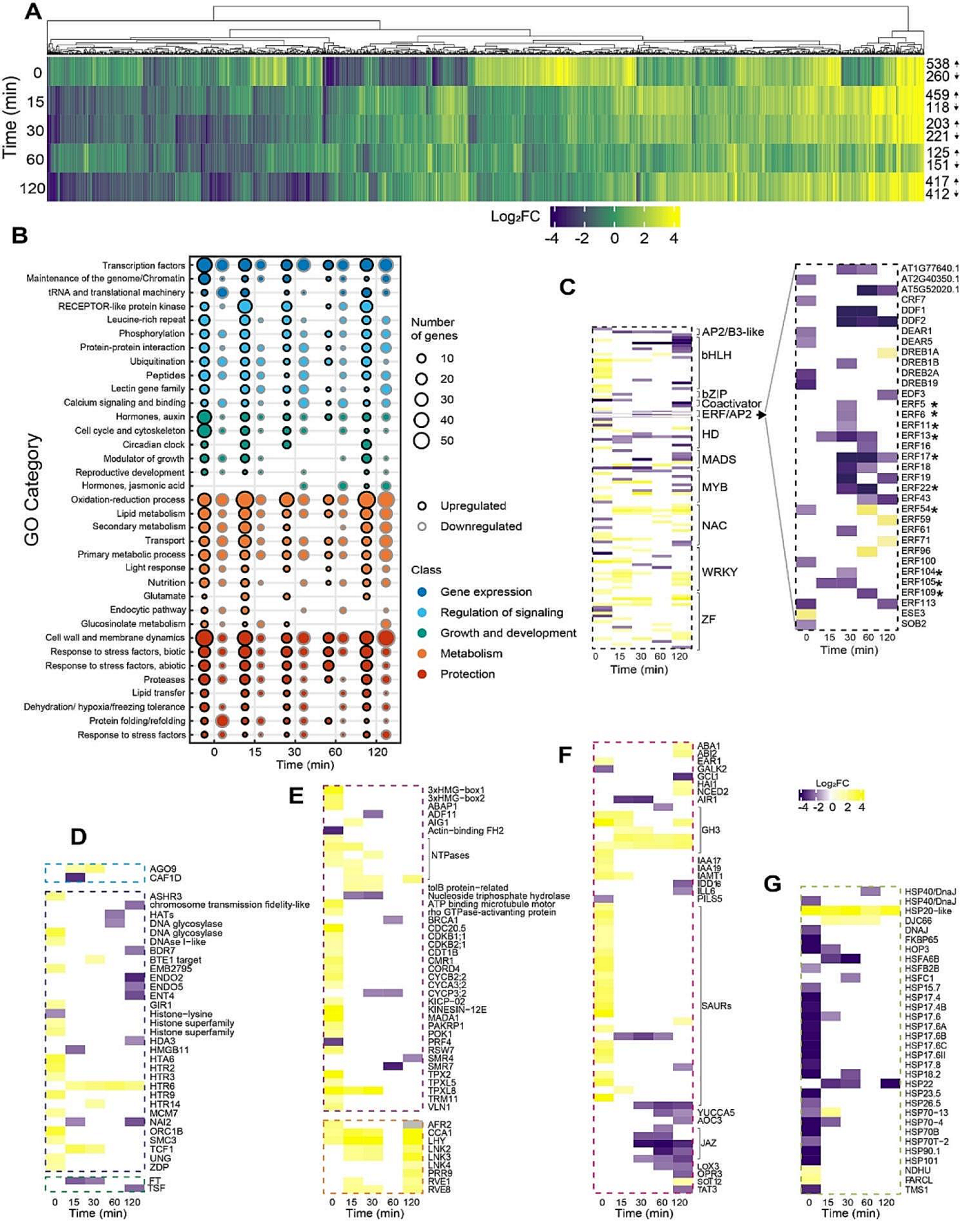
Comparison of WT and *atl80* transcriptomes in response to WDS. **(A)** Heat map displaying 1,938 DEGs at time points 0, 15, 30, 60, and 120 min following WDS. The number of upregulated and downregulated genes at each time point is indicated to the right. **(B)** GO visualization of relevant categories across five classes. Gene clustering was performed using the K-means method with the Complex Heatmap package in Rstudio. Additional details for specific terms are shown in: **(C)** TFs with a further breakdown of ERF/AP2s, *involved in retrograde signaling [32]; **(D)** posttranscriptional regulation, maintenance, and chromatin organization; growth and development category; **(E)** cell cycle and cytoskeleton and circadian clock master regulators; **(F)** hormones; **(G)** the protein folding/refolding. Supplementary Table 1 display a comprehensive list of the genes included in each class and category

Next, DEGs were identified through a comparative analysis of the WT and *atl80* transcriptomes across various time points (Fig. 4A). Fold change values were calculated using log2-normalized data from replicate samples, with a significance threshold of P-value < 0.05 and an absolute log2 fold-change cutoff ≥ 1.7. The number and proportion of upregulated and downregulated genes varied across different time points. Specifically, higher numbers of DEGs were observed at 0 and 120 min, and distinct patterns of upregulation and downregulation were evident within 0 and 15 min, indicating that ATL80 had a significant impact on gene expression. A number of differences between WT and *atl80* were detected before WDS (Fig. 4B). Furthermore, several differences between the WT and *atl80* transcriptomes were detected even before the onset of WDS (Fig. 4B). Genes related to the signaling class, including receptor-like proteins and leucine-rich repeat proteins, exhibited a general trend of upregulation both before and after WDS. In contrast, genes involved in phosphorylation, protein-protein interactions, ubiquitination, and peptides showed an overall trend of upregulated expression before WDS and at 15 min, followed by downregulation at 30, 60, and 120 min post-WDS. Similarly, genes related to metabolism and the protection class displayed a similar pattern of upregulated expression before WDS and after 15 min (Fig. 4B).

Although TFs were similarly regulated before and after WDS, they exhibited upregulation before WDS and significant downregulation after WDS (Fig. 4C). In contrast, WRKY, NAC DOMAIN CONTAINING PROTEIN (NAC), and zinc finger factors generally showed upregulation, while Ethylene-Responsive Factor/APETALA2 (ERF/AP2), MADS-box (MADS), and homeobox factors were predominantly downregulated. Basic helix-loop-helix (bHLH), Basic leucine-zipper (bZIP), and MYB domain proteins (MYBs) exhibited upregulation preceding WDS but were largely downregulated after the stress (Fig. 4C). NAC, MADS, homeobox, bHLH, and bZIP transcription factors are known to play diverse roles in growth and developmental processes (Supplementary Table 1). Conversely, other types of transcription factors primarily function in regulating stress responses. Several WRKY transcription factors, such as WRKY17, WRKY18, WRKY48, WRKY50, WRKY51, and WRKY70, are involved in plant immunity) [23]. Zinc finger factors like ZAT12 and ZAT18 are associated with responses to oxidative stress and drought stress tolerance [24, 25]. ERF/AP2 TFs play crucial roles in responding to abiotic stresses. For example, DREB2A and DREB19 respond to WDS, DDF1 and DDF2 are involved in gibberellic acid-mediated stress responses, and ERF96, ORA47, DREB1A/CBF3 participate in ABA-mediated responses to various stresses [26–31]. Furthermore, eleven ERF/AP2 TFs, including ERF022, ERF025, ERF017, ERF013, ERF011, ERF054, ERF6, ERF5, ERF105, ERF104, and ERF109/RRTF1, respond rapidly to high (H)-light and are involved in a retrograde signaling pathway [32]. In addition, MYC2 plays a significant role in JA-regulated signaling for stress responses and plant growth and development, while AITR2 is involved in the feedback regulation of ABA signaling [33, 34].

Before WDS, genes in the maintenance and chromatin organization category showed upregulation. This included histone superfamily genes, DNA glycosylases, origin of replication complex protein 1B, and condensing complex subunit, among others (Fig. 4D). Notably, the expression of *ARGONAUTE9* (*AGO9*), known for its role in silencing transposable elements, as well as several stress response genes, were upregulated at both 15 and 60 min. Additionally, *CAF1D*, a component of the deadenylase complex, exhibited downregulation at 15 min [35, 36].

Genes associated with growth and development also displayed upregulation prior to WDS. Two phosphatidylethanolamine-binding protein (PEBP) genes involved in reproductive development, namely *FLOWERING LOCUS T* (*FT*) at 15 and 30 min and *TWIN SISTER OF FT* (*TSF*) at 120 min, were found to be downregulated (Fig. 4D,E) [37]. Moreover, genes related to the cell cycle and cytoskeleton exhibited upregulation before WDS. The circadian clock master regulators *CCA1* and *LHY1* were upregulated at all time points except at 60 min; after 15 min of WDS, other regulators and clock-regulated genes, including *RVE1*, *RVE8*, *PRR9*, *LNK2*, and *LNK3*, displayed upregulation (Fig. 4E) [38, 39]. Additionally, genes regulated by auxin were upregulated prior to WDS, while those related to jasmonic acid (JA) showed downregulation after 30 min (Fig. 4F). Conversely, genes associated with protein folding/refolding were downregulated prior to WDS (Fig. 4G). It’s worth noting that cytosolic chaperones from the HSP90 and HSP70 families are known to regulate retrograde signaling [40].

### Effect of ATL80 on transcriptional responses induced by WDS

**Fig. 5:**
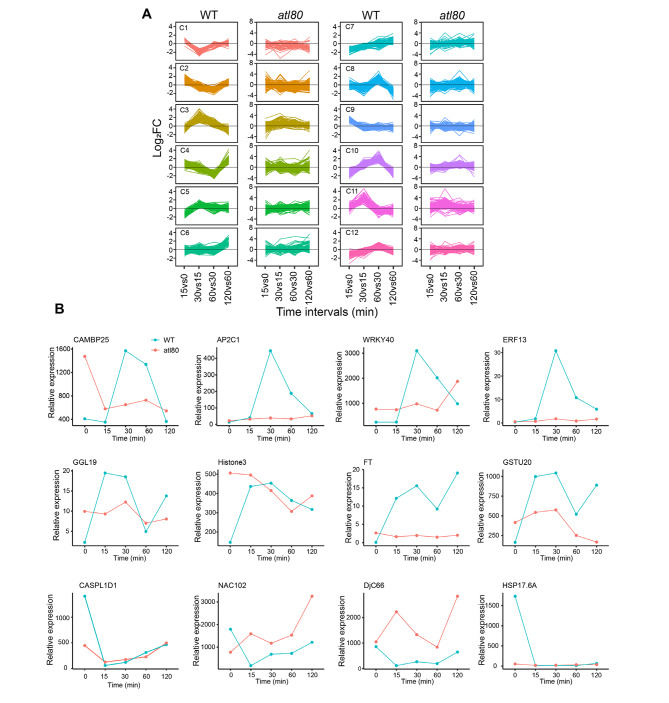
Patterns of rapidly induced transcription in response to WDS are abolished in *atl80*. **(A)** K-means cluster analysis of 2156 (DEGs) between time points in WT transcriptomes. DEGs log2FC values were extracted from the *atl80* mutant transcriptomic data and clustered by the K-means method. Cluster 11 includes *ATL80*. **(B)** Comparison of relative expression between WT and *atl80* in response to WDS, assessed by qRT-PCR analysis. *CAMBP25*, *AP2C1*, *WRKY40* and *ERF13* are representative genes selected from cluster 11, which contains 77 PRGs. *GGL19*, *Histone3*, *FT*, *GSTU20*, *CASPL1D1*, *NAC102*, *DJC66* and *HAP17.6 A* represent genes from other clusters

Previous studies exploring early responses to abiotic stresses have revealed a wide array of distinct gene expression patterns in reaction to stress exposure. In our investigation, we assumed the existence of multiple transcriptional waves triggered by WDS. To examine this, we utilized fold-change values to group genes based on their fold-differences, ultimately identifying 2,156 genes that displayed differential expression in at least one of the comparisons between consecutive time points during our time-course experiment. Subsequently, we organized these WDS-responsive genes into 12 distinct clusters. The expression values of each cluster relative to the *atl80* mutant were extracted from the transcriptomic data and clustered accordingly (Fig. 5A, Supplementary Table 2). Our primary objective was to ascertain whether ATL80 exerts an influence on the overall expression of WDS-responsive genes. Remarkably, the comparison of these twelve sets of clusters demonstrated that the expression patterns of WDS-responsive genes were predominantly reliant on ATL80, as the clustering patterns were disrupted in the *atl80* mutant. Additionally, qPCR analysis of PRGs and other genes responsive to WDS highlighted that ATL80 played a pivotal role in regulating these genes, as evidenced by the disruption of expression in the *atl80* mutant (Fig. 5B).

### Primary response genes among the genes responsive to WDS

**Fig. 6:**
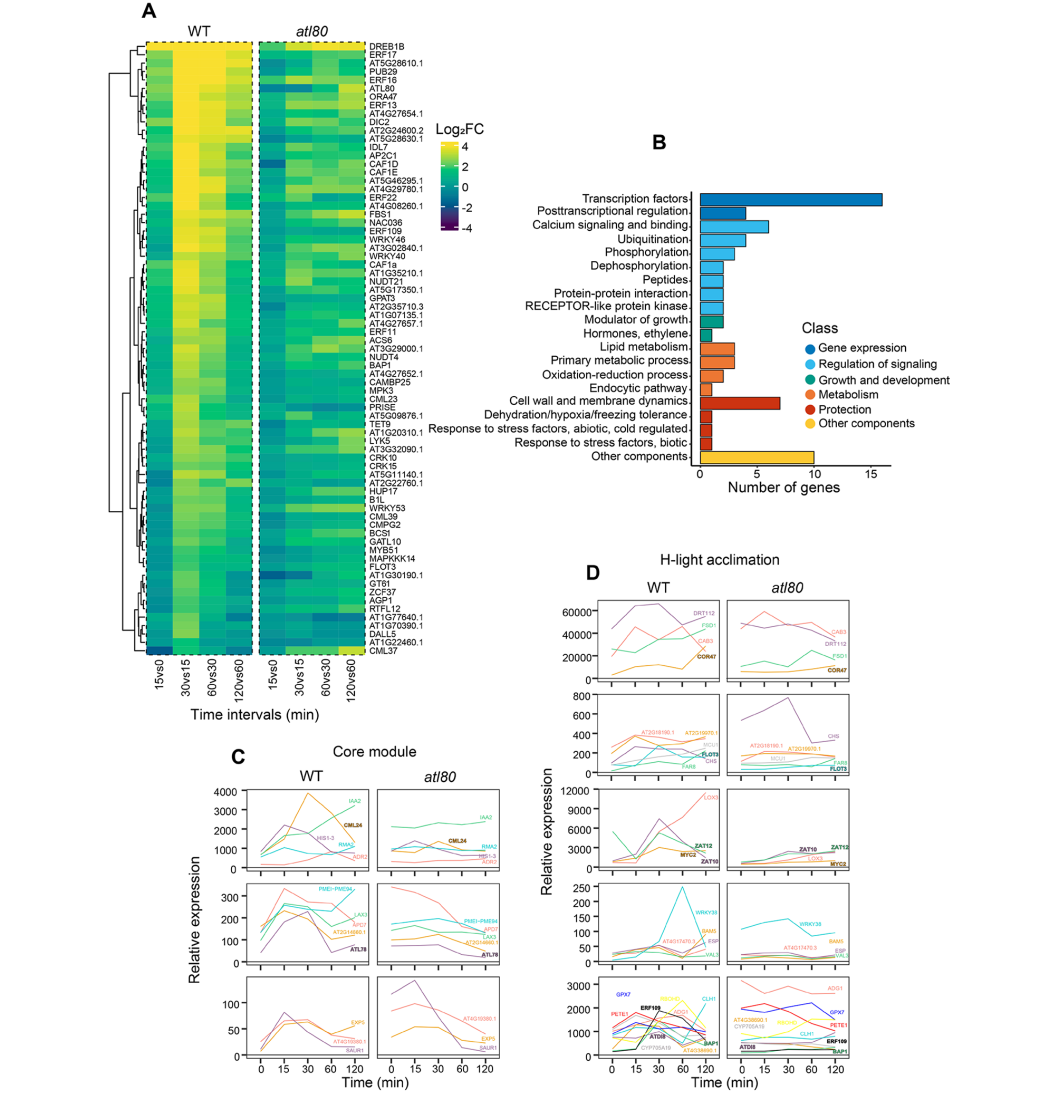
Primary response genes pattern in response to WDS. **(A)** Heat map displays the expression data of 73 PRGs in response to WDS, along with their respective expression values extracted from transcriptomic data of the *atl80* mutant. The 73 genes were specifically chosen from Cluster 11, which comprises a total of 129 genes, due to their pronounced similarity to the expression pattern observed in ATL80. **(B)** GO analysis of the 73 PRGs. **(C)** Cross-reference of transcriptomic data related to core response module of retrograde signaling pathway genes with the ATL80 transcriptome. The mean expression of 13 genes is illustrated in three graphs based on expression intensity and is compared to their respective expression levels in the *atl80* mutant (Supplementary Table 3). **(D)** Cross-reference of transcriptomic data related to genes responding to H-light acclimation. The mean expression of 29 genes is depicted in five graphs based on expression intensity and is compared to their corresponding expression in the *atl80* mutant (Supplementary Table 3)

Among the genes responsive to WDS, we have identified a subset of PRGs. These PRGs exhibit a transient induction of expression, peaking at 30 min following WDS. To gain insights into the regulation and function of ATL80, we focused on the subset of 73 PRGs that closely match the expression pattern of *ATL80 *among PRGs with this transient induction. Within this group of 73 WDS-responsive PRGs (as depicted in Fig. 6A), we performed Gene Ontology (GO) term enrichment analysis (Fig. 6B). Notably, we observed the presence of ERF/AP2 and WRKY transcription factors (TFs), with 10 members belonging to the ERF/AP2 family and 4 to the WRKY family. Among the ERF/AP2 TFs, *OCTADECANOID-RESPONSIVE AP2/ERF 47* (*ORA47*) is known for facilitating a rapid response to environmental signals. *C-REPEAT*/*DRE BINDING FACTOR1* (*CBF1*) plays a critical role in cold acclimation, while *REDOX RESPONSIVE TRANSCRIPTION FACTOR1* (*ERF109*/*RRTF1*) is involve in several stress responses and integrates age and wound signals for root regeneration [41–43]. On the other hand, *WRKY40* is primarily involved in perceiving and responding to biotic stress, *WRKY46* is associated with drought stress tolerance, and *WRKY53*, along with *WRKY18*, activates early expression of several sugar-responsive genes [44, 45]. In the posttranscriptional regulation category, we found *CCR4-ASSOCIATED FACTOR1* (*CAF1A*), *CAF1D*, and *CAF1E*, which are orthologs of the yeast deadenylase component CAF1 [36].

In the regulation of signaling category, two key genes are *CALMODULIN LIKE 37* (*CML37*), which plays a crucial role in defense responses under stress conditions and *CAMBP25*, which acts as a negative effector of osmotic stress tolerance [46–48]. Additionally, *CML39* is involved in various developmental processes and has a role in light-promoted seedling development [49, 50].

In the phosphorylation category, various genes are highlighted. *LYSM-CONTAINING RECEPTOR-LIKE KINASE 5* (*LYK5*) is crucial for chitin perception [10, 51, 52]. AP2C1, which is closely related to PROTEIN PHOSPHATASE 2C5 and interacts with stress-induced Mitogen-Activated Protein Kinases (MAPKs) MPK3, MPK4, and MPK6. AP2C1 functions as a MAPK phosphatase, regulating ABA-induced gene activation [53]. *MPK3* and *MPK6* are PRGs that enhance ethylene biosynthesis in response to wounding, and *MAP3K14*, whose expression is rapidly induced in response to wounding, is part of two modules that rapidly respond to wounding [54].

In the hormone category, *1-AMINOCYCLOPROPANE-1-CARBOXYLIC ACID SYNTHASE 6* (*ACS6*) is a member of the ACC synthase family of ethylene biosynthetic enzymes. Its activation is involved in regulating stomatal density on the leaf epidermis in response to drought [55]. Additionally, *F-BOX STRESS INDUCED 1* (*FBS1*) influences the expression of genes regulated by JA and abscisic acid [56–58]. Several genes involved in biotic and abiotic stresses were also share a primary response genes pattern. *BYPASS1-LIKE* (*B1L*) regulates growth and cold tolerance [59–63]. Besides, *FLOTILLIN1* (*FLOT1*) and *FLOT2* are genes that form membrane nanodomains, potentially assisting in communication between the plasma membrane and the extracellular environment [64].

Convergence among retrograde signaling pathways and the influence of ATL80 on gene expression were deduced in our previous analysis (Fig. 4C and G). To provide support to such assumption, we cross-referenced data associated with retrograde signaling pathways against the early ATL80 transcriptome. We employ a set of 39 genes that form a core response module for retrograde signaling, along with a set of 64 reference genes analyzed in an experiment on conditional acclimation following six hours of H-light treatment [65, 66]. We investigated whether among them they were WDS-responsive genes. Thirteen genes were identified within the 39 core response genes, while 29 were detected in the 64 genes responsive to H-light (Fig. 6C and D) (Supplementary Table 3). The essential regulatory role of ATL80 in early expression of several these genes is evident, as demonstrated by the observed alteration in expression in the *atl80* mutant (Fig. 6C and D; left graphs).

Among the genes involved in retrograde signaling pathways, six TFs exhibited increased expression early in response to WDS. Notably, ERD109/RRTF1 has roles in both abiotic and biotic stress responses (Fig. 6D, lower graphs) [43]. Additionally, MYC2 serves as a key regulator of the JA signaling, impacting stress responses and plant growth and development (Fig. 6D, middle graphs) [67]. ZAT10 and ZAT12, are implicated in diverse stress responses, including drought, H-light, salt, cold, oxidative stress and osmotic stress (Fig. 6D, middle graphs) [68]. In the chromatin organization category, HIS1-3 encode a histone variant whose expression is induced by drought, salt and ABA (Fig. 6C, upper graphs) [69].

Within the calcium signaling and binding category, two pertinent genes are CML24 whose expression is enhanced in response to environmental changes, hydrogen peroxide, ABA, and IAA (Fig. 6C, upper graphs) [70]. Furthermore, BAP1, in conjunction with BON1 control plant growth homeostasis. BON1 proteins regulate global stress responses, including calcium signaling (Fig. 6D, lower graphs) [71]. Remarkably, COR47 and ATDI8, members of the dehydrin family that share highly conserved structural features (Fig. 6D, upper and lower graphs respectively), along with the RING-H2 E3 ubiquitin ligase ATL78 that has a role in adaptation to drought tolerance and FLOT3 found in membrane nanodomains, are also present among the cross-referenced genes (Fig. 6C, middle graphs; Fig. 6D, second graphs respectively) [17, 64, 72].

### Enrichment for calmodulin-binding transcription activator (CAMTA) TFs binding sites in the PRG promoters

**Fig. 7:**
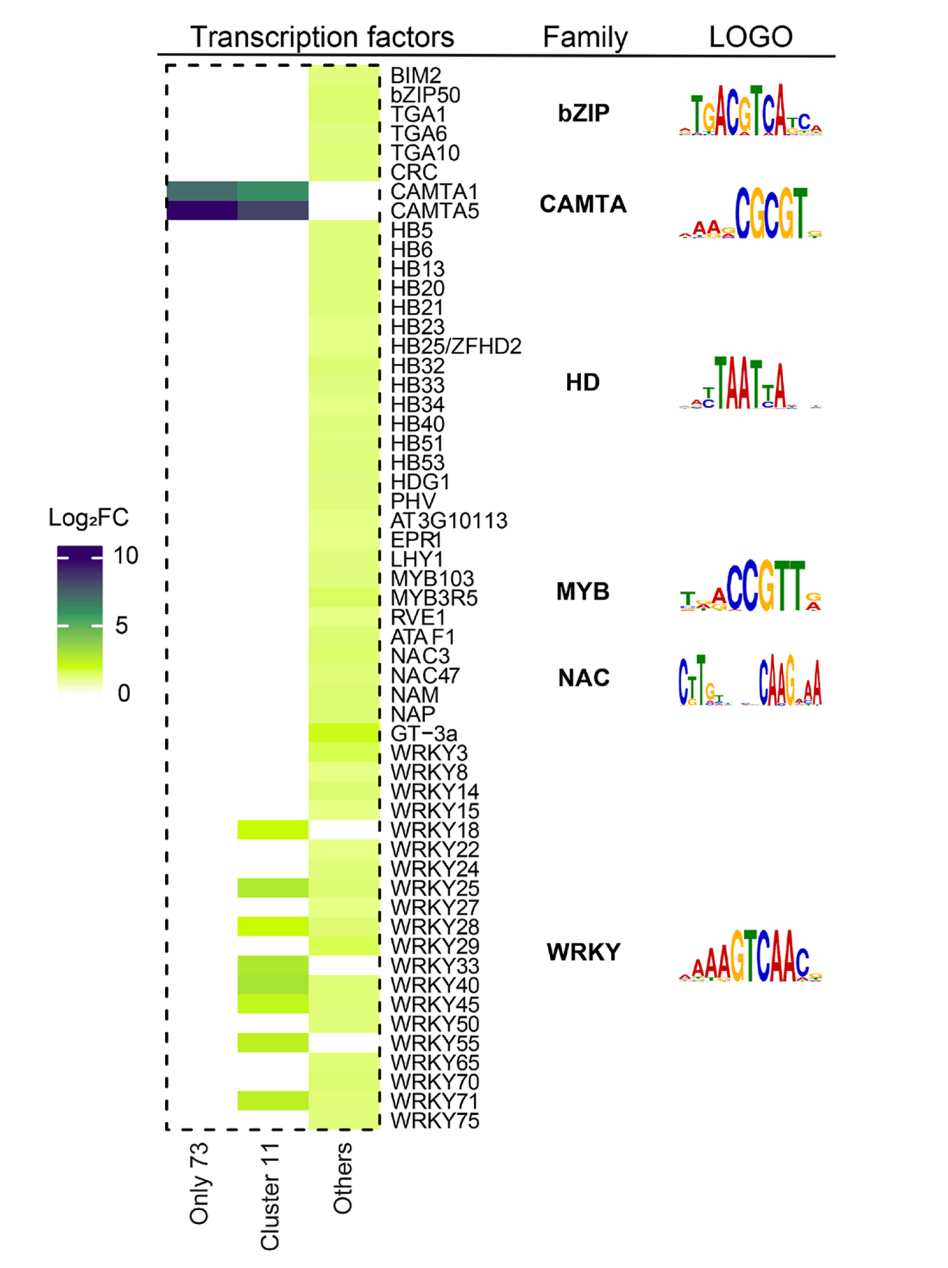
Enrichment analysis of cis-regulatory elements on PRGs. Heat map displaying the results of the cis-regulatory elements enrichment analysis of PRGs. This analysis was performed using the CisCross web service with default parameters. TF types are listed along with the family and representative sequence features for each type. The 73 PRGs were analyzed independently or together with the 129 genes in Cluster 11. “Other” refers to genes from the rest of the clusters. The respective logos were downloaded from the website http://neomorph.salk.edu/dap_web/pages/index.php [20]. The analysis was conducted independently for the 73 PRGs, collectively for the 129 genes in Cluster 11 that include the 73 PRGs, and as the group for genes from the rest of the clusters labeled as “Other”

TFs play a pivotal role in the initial control of gene expression by binding to specific DNA sequences, typically located in gene promoter regions. These DNA sequences in the promoters are specific cis-regulatory elements (CREs), which, on a genome-wide scale, collectively form the cistrome. To examine the potential association between TFs to cis-regulatory elements using the CisCross tool which predicts upstream regulators [19]. We evaluated the enrichment of cis-elements on the 73 set of PRGs and compared this the enrichment with cluster 11, the originally cluster that included 129 genes, and to 2027 WDS-responding genes out of this cluster (Fig. 7).

We then compare the enrichment of TFs to cis-regulatory elements between PRGs and those that do not exhibit a primary response gene pattern (Fig. 7). Our analysis revealed the presence of TFs exclusively enriched in PRGs, indicating their potential involvement in the regulation of these genes. Particularly noteworthy was the exclusive enrichment of binding sites for CALMODULIN-BINDING TRANSCRIPTION ACTIVATOR (CAMTA) TFs. CAMTA TFs are known to play a crucial role in the rapid response to a wide range of stresses. Specifically, CAMTA1 has been implicated as a regulator of drought stress, while CAMTA5 is rapidly activated in response to an immediate decrease in temperature, where it activates the expression of *DREB1* genes [73–75]. Our analysis also revealed the enrichment of several WRKY TFs when all genes from cluster 11 were included. Furthermore, we observed a significant assortment and enrichment of TFs among other WDS-responsive genes, highlighting a diversity of TFs enriched among the genes in the other clusters (bZIP, HD, MYB, NAC) (Fig. 7).

## Discussion

Primary response genes serve as key regulators in the transition of short-lived signals into prolonged responses. Their expression is rapidly triggered within minutes of perceiving stimuli, reaching its peak at around 60 min before gradually declining. The swift transcriptional responses to water deficit stress at brief time intervals imply that these responses may play a pivotal role in enabling plants to adapt to such challenging conditions. Despite ATL80 being characterized as an E3 ubiquitin ligase, typically associated with post-translational regulation, our hypothesis posits that, as a PRG, it might have a significant influence on gene expression. Our findings provide evidence that ATL80 indeed plays a critical role in modulating early responses to WDS, as demonstrated by the observation that gene expression within the 0-120 min time frame was profoundly disrupted in *atl80* mutant plants. This assertion is further supported by the presence of numerous DEGs between the WT and *atl80* mutant, many of which are known to have substantial roles in stress responses or response to WDS. Notable examples include several *WRKY* genes, *ZAT12*, *ZAT18*, *DREB2A*, *DREB19*, *DDF1*, *DDF2*, *ERF96*, *ORA47*, and *DREB1A*/*CBF3* (Fig. 4). For instance, *WRKY46* has been implicated in drought tolerance, as demonstrated by loss-of-function mutants that revealed its importance in this context [44, 45]. Additionally, the ectopic expression of *ERF019*, *DREB2A*, or *DDF1* has been shown to confer significant drought stress tolerance and activate various genes involved in the response to WDS [26–28] (Fig. 4).

The analysis of transcriptome data at various time points following WDS revealed a significant reduction (up to 95%) in the number of DEGs in the *atl80* mutant compared to the WT, indicating that ATL80 plays a pivotal role in modulating gene expression during the initial two hours after WDS (see Fig. 3b). It is conceivable that ATL80 may also exhibit peaks or additional expression patterns at extended time intervals in response to WDS (beyond several hours). For example, ZAT12 and WRKY40 transcription factors have been shown to display three distinct expression patterns at seconds, minutes, and hours in response to stress [76]. The presence of such expression patterns suggests that bursts of regulatory gene expression exert a significant influence on the transcriptomic landscape, contributing to the shaping and fine-tuning of the stress response.

In the absence of stress, ATL80 exerts an influence on the expression of several classes of genes. These genes span various functional categories, with upregulated genes related to the maintenance of genome/chromatin organization, remodeling, growth and development, cell cycle, cytoskeleton, and genes involved in auxin, brassinosteroids, cytokinin, gibberellic acid, and ethylene metabolism. Conversely, genes categorized under protein folding/refolding showed a downregulation, including notable chaperones like HSP90.1, DNAJ-domain proteins, HSP70, HSP17, and HSP20-like chaperones. These molecular chaperones play pivotal roles in diverse growth and plant development processes, facilitating the proper folding of polypeptide chains to prevent protein aggregation during stress responses [77–79]. Following exposure to WDS, the expression of these genes was no longer differential, suggesting a rapid transcriptome reprogramming in response to stress. Similar swift responses have been previously documented, where various genes displayed altered expression patterns within minutes or seconds in response to abiotic stresses [80, 81].

The rapid response to WDS results in the activation of calcium signaling. Calcium has also been associated with rapid, widespread signaling activity across the plant [82]. Among primary response genes to WDS included in the calcium signaling and calcium binding category genes, are regulators of the ABA pathway. Additionally, *CML37* is a calmodulin-like gene that functions as a positive regulator of ABA [47]. A search for general stress response regulators identified *ORA47*, a jasmonic acid-induced gene, and *JAZ1* which acts as a repressor may serve as a transcriptional regulator for a central hub controlling the general stress response [30]. The finding that several genes encoding JA precursor of biosynthetic proteins and signaling cascade components that are among the primary response genes identified here support such a mechanism that entails the interaction between the JA metabolism and the reprograming of stress responses.

In the subset of 73 PRGs, there are several regulatory genes implicated in stress responses, such as ERF/AP2 and WRKY TFs, calcium signaling genes, MAP kinases, and various signaling peptides associated with stress (Fig. 6). The expression of these specific genes may share common cis-regulatory elements, as indicated by the analysis of the promoters of these 73 PRGs, which predicts an enrichment of binding sites for CAMTA1 and CAMTA5. CAMTA TFs play a crucial role in rapidly responding to various stresses. Notably, CAMTA family genes have been studied in various plant species. CAMTA1 has been identified as a key regulator of drought stress in *A. thaliana* [73, 74]. Transcriptome data from *Heimia myrtifolia* revealed three upregulated CAMTA genes in response to WDS [83]. Similarly, *Phyllostachys edulis* encode CAMTA genes associated with drought stress, with promoter analysis indicating the presence of stress-related cis-elements [84]. *Camellia sinensis* displayed distinctive expression patterns of CAMTA genes across organs and diverse stress conditions [85]. Notably, studies in Quinoa have identified the role of *CqCAMTA03* in enhancing drought stress tolerance [86]. In wheat, *TaCAMTA1b-B.1* emerged as a participant in the drought stress response during the seedling stage [87]. Additionally, specific CAMTA genes in soybeans were identified as negative regulators influencing both development and responses to drought stress [88]. Additionally, it is worth noting that among the 73 PRGs are CAF1A, CAF1D, and CAF1E, which may be components of the deadenylase complex. These findings suggest a potential role in mediating the transient half-life of rapidly induced mRNAs, including PRGs, in response to WDS [36].

**Fig. 8:**
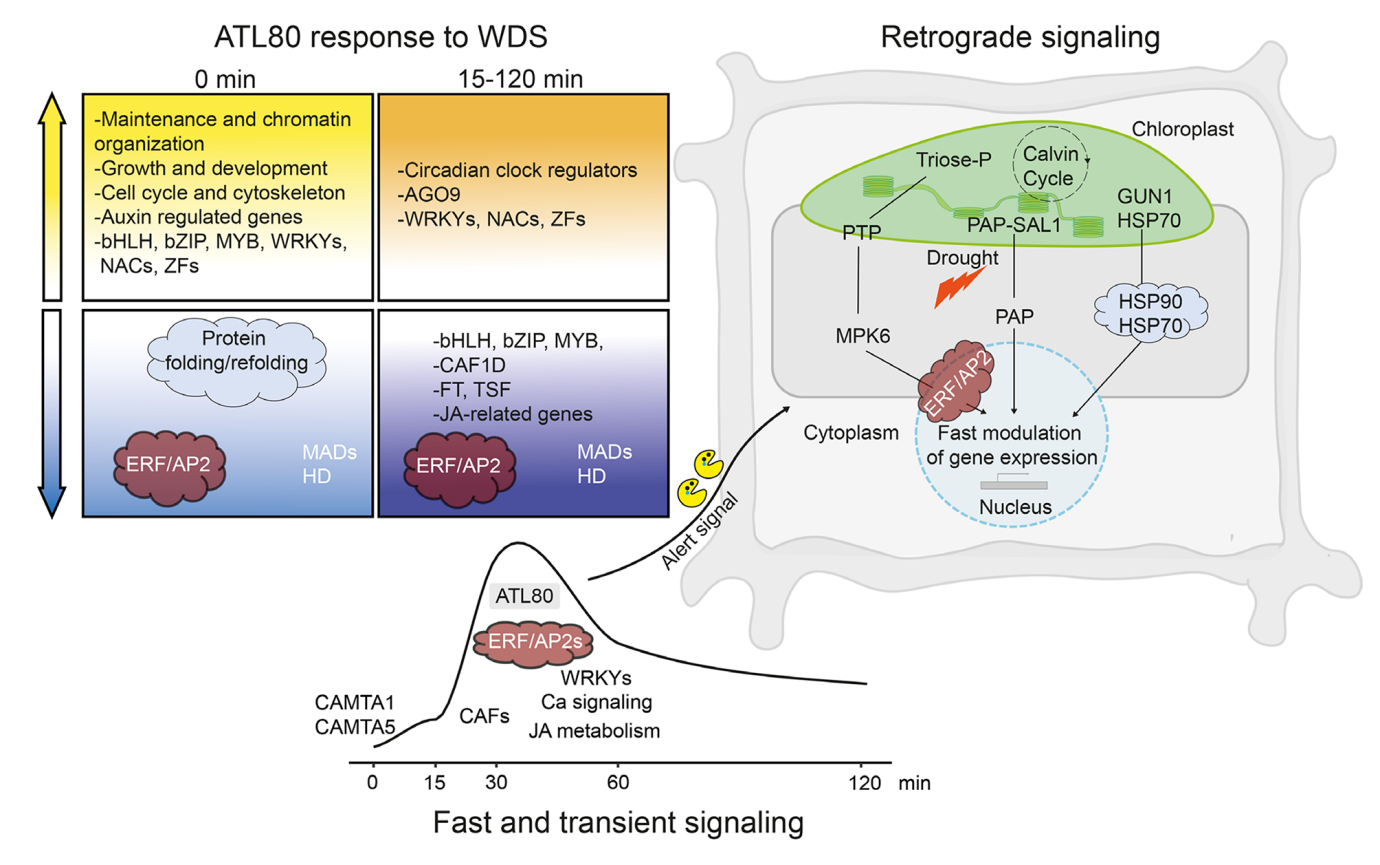
Schematic of the early ATL80-dependent response to WDS and its presumed function in retrograde signaling pathways. The *ATL80* response to WDS is depicted in two columns: one representing the condition before stress (0 min, light colors), and the other showing the response after 15 to 120 min following stress (darker colors). Upregulation and downregulation events are indicated by yellow and violet arrows, respectively. GO classes, categories, or individual genes are denoted. Below this representation, we illustrate the expression patterns of *ATL80* along with other PRGs. We postulate that CAMTA TFs are involved in regulating the expression of this set of PRGs. On the right side of the figure, we outline the basic retrograde signaling pathways. The pathway triggered by H-light exposure involves the translocation of triose phosphate, a product of the Calvin-Benson cycle, from the chloroplast through a triose phosphate translocator (TPT). This process activates the kinase MPK6 and ERF/AP2 TFs. The GUN1–HSC70 complex initiates signaling, which is mediated by cytosolic chaperones HSP90 and HSP70. The PAP-SAL1 signaling pathway responds to drought and H-light stress [90]. We have encircled components that were both detected as DEGs in our ATL80 transcriptome and are involved in retrograde signaling with a cloud shape (ERF/AP2 and proteins associated with folding/refolding/HSP90/HSP70). We hypothesize that one or more components of the retrograde signaling pathways (highlighted in light gray) serve as targets for degradation by ATL80, indicated by the arrow pointing toward the shaded region, functioning as an alarm signal

Among DEGs, 52 ERF/AP2 TFs were identified, with 44 of them showing gene expression fluctuations within 120 minutes after subjecting the plants to a H-light intensity. Previously, 19 ERF/AP2 TFs that responded rapidly when transitioning low-light-acclimated *A*. *thaliana* plants to H-light conditions had been identified. Notably, 11 of these ERF/AP2 TFs are among the DEGs. Upon exposure to H-light conditions, there is a rapid communication pathway from the chloroplast to the nucleus, known as retrograde signaling. This pathway swiftly conveys information that impacts gene expression in response to developmental signals and environmental stresses [32]. The rapid induction of a similar set of ERF/AP2s in response to both WDS and H-light suggests a convergence of signaling pathways (Fig. 8). GENOMES UNCOUPLED1 (GUN1), a protein localized in the chloroplast with a central role in retrograde signaling, interacts with ClpC1, a chloroplast chaperone associated with the protein import machinery. In a double *gun1clpc1* mutant, the expression of members from the HSP90 and HSP70 families is significantly increased (Fig. 8). This finding reveals a role for cytosolic HSP90-HSP70 chaperone complexes in retrograde signaling [40]. Genes encoding HSP90.1 and HSP70 chaperones exhibited downregulation in the *atl80* mutant prior to WD, suggesting a convergence of both signaling pathways. The early expression of several core module genes, identified through the analysis of transcriptional responses in mutant lines compared to WT lines across six different experiments impacting retrograde signaling, as well as a set of reference signaling genes derived from an independent experiment on the initiation of H-light treatment, substantiates the convergence of these pathways in response to WDS (Fig. 6C and D) [65, 66]. Another pathway with a role in retrograde signaling, responding to drought and H-light stress, is the SAL1-nucleotide 3’-phosphoadenosine 5’-phosphate (PAP) signaling pathway. Drought and H-light stress inhibit SAL1 activity, leading to an increase in PAP accumulation within the chloroplasts. PAP is proposed to serve as a retrograde signal that induces the expression of nuclear genes through a yet-unknown mechanism [89]. These observations highlight ATL80 as a potential candidate for bridging various retrograde signaling pathways (Fig. 8).

Our results suggest that ATL80 has a rapid and significant impact on gene expression, as evidenced by a remarkable reduction in DEGs between the time points 0 to 120 min during the time course experiment when comparing the *atl80* mutant to the WT. A similar rapid impact on gene expression is observed in response to H-light intensity. The expression of *ERF*/*AP2* TFs and *MITOGEN-ACTIVATED PROTEIN KINASE6* (*MPK6*) genes is highly induced within minutes. Notably, this rapid response is disrupted in the triose phosphate/phosphate translocator 1 *(tpt1*) and *tpt2* mutant lines, implying that TPT plays a crucial role in early transcriptional responses to H-light. TPT facilitates the translocation of triose phosphate from the chloroplast, constituting a retrograde signaling pathway [32]. Furthermore, our findings suggest that components of the retrograde signaling pathway, which regulate transcriptional responses within minutes, consist of proteins other than TFs. This hints at the possibility that the E3 ubiquitin ligase ATL80 may target one or more of these components.

## Conclusion

To maintain cellular homeostasis under stressful conditions, transcriptional reprogramming plays a critical role. Reprogramming of the transcriptome can happen within seconds following a cellular stressor (i.e., WDS), initiating a rapid response that can activate both well-known and previously unrecognized proteins and pathways. Chloroplasts are capable of sensing drought stress and generating retrograde signals that subsequently regulate the cellular stress response. Several components of retrograde signaling serve the function of modulating transcription, such as TPT. ATL80 is an E3 ligase primarily localized to the plasma membrane [22]. Our results suggest that ATL80 has a positive impact on gene expression when responding to WDS. We propose that ATL80 plays a role in the early response to WDS by reducing the half-life of components within retrograde signaling pathways, acting as an E3 ligase. Furthermore, it is possible that ATL80 might exhibit varying peaks or show additional expression patterns at extended time intervals following exposure to WDS (lasting for more than a few hours). For instance, certain PRG TFs exhibit three distinct expression patterns, which occur within seconds, minutes, and hours in response to stress [76]. The presence of this expression pattern indicates that bursts in regulatory gene activity impact the transcriptomic landscape of the plant, shaping and fine-tuning the stress response. This, in turn, modulates distinct retrograde signaling pathways, serving as an early alarm signal to mitigate damage during the initial stages of the stress response (Fig. 8).

### Electronic supplementary material

Below is the link to the electronic supplementary material.

Supplementary Material 1: Table 1. Differentially expressed genes between the *atl80* mutant and the WT transcriptomes at different time points.

Supplementary Material 2: Table 2. Gene expression rapidly induced in response to water deprivation stress compiled in twelve clusters.

Supplementary Material 3: Table 3. Cross-reference of transcriptomic data related to retrograde signaling pathways with the ATL80 transcriptome.

## Data Availability

The authors declare that all data supporting the findings in this study are available within the paper and Supplementary information. All data are available upon request to the corresponding author.

## Data availability

The authors declare that all data supporting the findings in this study are available within the paper and Supplementary information. All data are available upon request to the corresponding author.

## Abbreviations

PRG: Primary response gene
WDS: Water deprivation stress
TF: Transcription factor
